# Real‐World Treatment Patterns and Outcomes in Patients With Relapsed/Refractory Multiple Myeloma and 1–3 Prior Lines of Therapy: Optum Database

**DOI:** 10.1002/cam4.71093

**Published:** 2025-07-28

**Authors:** Binod Dhakal, Hermann Einsele, Jordan M. Schecter, William Deraedt, Nikoletta Lendvai, Ana Slaughter, Carolina Lonardi, Sandhya Nair, Nirosha Elsem Varghese, Jianming He, Akshay Kharat, Seina Lee, Patricia Cost, Ravi Potluri, Mythili Koneru, Nitin Patel, Erika Florendo, Paula Rodriguez‐Otero, Kwee Yong

**Affiliations:** ^1^ Medical College of Wisconsin Milwaukee Wisconsin USA; ^2^ Universitätsklinikum Würzburg Medizinische Klinik und Poliklinik II Würzburg Germany; ^3^ Johnson & Johnson Raritan New Jersey USA; ^4^ Johnson & Johnson Beerse Belgium; ^5^ Cilag GmbH International Zug Switzerland; ^6^ Johnson & Johnson Buenos Aires Argentina; ^7^ Johnson & Johnson Horsham Pennsylvania USA; ^8^ Putnam Associates New York New York USA; ^9^ Legend Biotech USA Inc. Somerset New Jersey USA; ^10^ Clínica Universidad de Navarra CIMA, CIBERONC, IDISNA Pamplona Spain; ^11^ University College London Cancer Institute London UK

**Keywords:** health care resource utilization, multiple myeloma, real‐world outcomes, refractory

## Abstract

**Background:**

Early (often continuous) treatment of multiple myeloma (MM) with lenalidomide has become common practice, leading to an increase in lenalidomide‐refractory disease.

**Methods:**

We report real‐world treatment patterns, health care resource utilization (HCRU), and outcomes for patients with lenalidomide‐refractory MM using data from Optum US Claims and Optum electronic health record (EHR) databases with index date from January 2016 to March 2022 (Claims) or December 2021 (EHR). Eligible patients had received 1–3 prior lines of therapy (LOT), including a proteasome inhibitor.

**Results:**

A total of 1383 and 1597 patients with lenalidomide‐refractory disease were included from the Claims and EHR databases, respectively, with median ages of 72 and 68 years and mean Charlson Comorbidity Index scores of 4.0 and 3.1. The most common treatment combinations were daratumumab–pomalidomide–dexamethasone, daratumumab–bortezomib–dexamethasone, and pomalidomide–dexamethasone (~5% each). From LOT 2 to LOT 6, treatment attrition (patients who died or received no further treatment) was 95.2% to 95.9%. Median time to next treatment was 5.4 (Claims) and 5.9 months (EHR). Median OS was 35.2 (Claims) and 41.2 months (EHR). HCRU was consistent across LOT.

**Conclusions:**

Patients with lenalidomide‐refractory MM who received 1–3 prior LOT had poor outcomes and moved quickly through available therapies, demonstrating an unmet need to improve outcomes in this difficult‐to‐treat patient population.

## 1 Introduction

Lenalidomide‐based regimens are recommended by the National Comprehensive Cancer Network (NCCN) Clinical Practice Guidelines in Oncology (NCCN Guidelines) and the European Society for Medical Oncology for the initial treatment of all patients with multiple myeloma (MM), including those both eligible and ineligible for transplant [1, 2]. After this initial therapy, many patients are maintained on lenalidomide until their disease progresses. The prominence of lenalidomide‐based frontline therapy, as well as its use in relapsed/refractory disease, has resulted in a growing population with lenalidomide‐refractory disease [3]. Despite a range of subsequent treatment options, including doublet and triplet combinations of proteasome inhibitors (PIs), immunomodulatory drugs, and anti‐CD38 monoclonal antibodies, the median progression‐free survival (PFS) reported in clinical trials in lenalidomide‐refractory MM is generally < 12 months [3, 4, 5, 6, 7]. Furthermore, increasing refractoriness to these drug classes predicts poorer prognosis [8].

Older patients and those with substantial comorbidity burdens are often underrepresented in randomized, controlled trials [9, 10]. For example, renal impairment is a widely used exclusion criterion in randomized, controlled trials and a well‐known prognostic factor [10]. There is a paucity of information on treatment patterns, health care resource utilization (HCRU), and outcomes for real‐world populations of patients with lenalidomide‐refractory relapsed/refractory (RR) MM since the emergence of newer triplet combinations, and the best regimens for use in clinical practice for these difficult‐to‐treat patients are unclear. Evidence is needed to provide insights into how treatments that have come into regular use in the past few years may perform in lenalidomide‐refractory disease in clinical practice.

To investigate unmet need, we report the treatment patterns, HCRU, and outcomes for patients with MM who have received one to three prior lines of therapy (LOT), including a PI and lenalidomide, with a focus on disease that is refractory to lenalidomide. This population aligns with that of the CARTITUDE‐4 trial of ciltacabtagene autoleucel (cilta‐cel) chimeric antigen receptor T‐cell (CAR‐T) therapy, which was approved for use in patients with lenalidomide‐refractory disease and ≥ 1 prior LOT in April 2024. Some data on lenalidomide‐sensitive disease have been provided for context. The analysis was limited to data from 2016 to 2022 to concentrate on outcomes following the introduction of recent treatments, including daratumumab.

## 2 Materials and Methods

### 2.1 Databases

Data were derived from Optum Clinformatics Data Mart US Claims (data collection range: January 2010 to March 2022) and Optum electronic health records (EHRs; January 2010 to December 2021). Optum Clinformatics Data Mart is a de‐identified administrative health claims database from commercial and Medicare Advantage health plans, representing members from all 50 states in the United States. The Optum EHR is a longitudinal repository derived from > 700 hospitals and > 7000 clinics across the United States. Data analysis was restricted to January 2016 onward to reflect the current treatment landscape.

### 2.2 Patients and Treatments

Adult patients with MM who had received 1–3 prior LOT and were exposed to a PI and lenalidomide were included. The patients were categorized as having lenalidomide‐refractory or lenalidomide‐exposed‐nonrefractory disease at the time all inclusion criteria were met. Lenalidomide‐refractory was defined as having a change in treatment within 60 days of the last lenalidomide therapy without lenalidomide as a component of the immediate next line, as previously described [11]. The lenalidomide‐exposed‐nonrefractory population included patients who had previously received lenalidomide therapy for > 60 days or had received lenalidomide as a component of the immediate next line at the time they met all other inclusion criteria and were not classified as lenalidomide‐refractory [11]. Treatment pattern analysis provided for gaps of up to 180 days in treatment to be bridged, so that longer LOT in which the drug regimen was unchanged or reduced to a subset of the same drugs were considered a single LOT.

### 2.3 Analyses

Time zero (T0), or index date, was defined as the start date of first subsequent treatment (index therapy) post eligibility. Baseline characteristics and treatment patterns were assessed using descriptive statistics. The attrition rate was analyzed in patients who had received 1 prior LOT and was defined as the proportion of patients without a record of subsequent MM treatment who were lost to follow‐up or died. Patients in a treatment‐free interval before the next LOT are not counted in the attrition rate. Cumulative attrition rates were calculated using the number of patients who received the first LOT after meeting inclusion criteria as the denominator.

The Kaplan–Meier method was used to estimate survival outcomes (overall survival [OS], time to next treatment [TTNT], and time to treatment discontinuation [TTTD]), starting at T0 (index date). OS was defined as the time interval between the index date and all‐cause mortality. TTNT was defined as the time interval between the index date and the end of that treatment or death, whichever came first. TTTD was defined as the time interval between the index date and either the end of that treatment or death, or if the interval from the end of treatment to loss to follow‐up was > 120 days. The log‐rank test for trend was applied to compare time‐to‐event data in patients with 1 versus 2 versus 3 prior LOT.

HCRU was analyzed in the Claims database only, as it more effectively captures treatment history compared with EHRs. Outpatient visits, hospital stays, emergency visits, and laboratory visits were summarized with descriptive statistics. These were described within the time period defined as the start of a given LOT until 1 day before the start of the next LOT. If the LOT was the final LOT recorded for a patient, the time period ended at the end of the LOT plus 90 days or loss to follow‐up, whichever occurred first.

## 3 Results

### 3.1 Patients With Lenalidomide‐Refractory Disease

**FIGURE 1:**
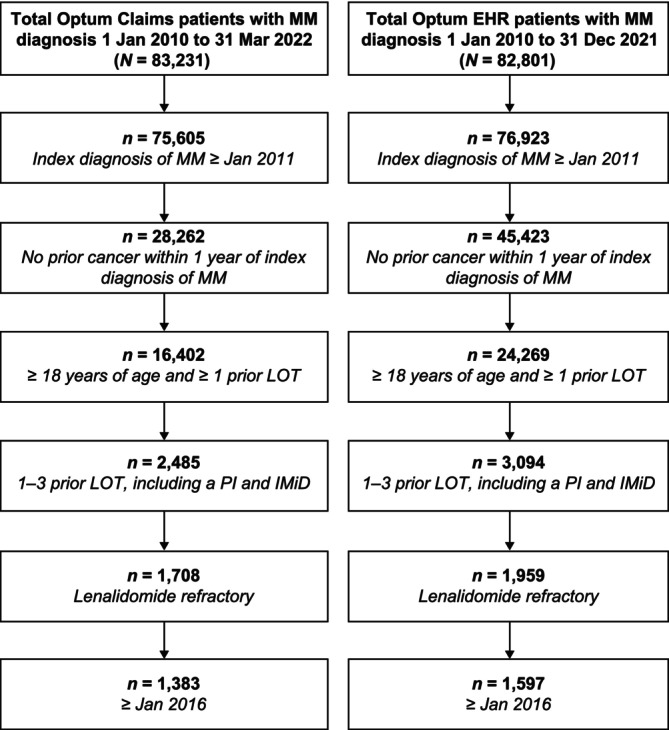
Patient selection. EHR, electronic health records; IMiD, immunomodulatory drug; LOT, lines of therapy; MM, multiple myeloma; PI, proteasome inhibitor.

From 83,231 total Claims patients with a diagnosis of MM, 1383 patients with lenalidomide‐refractory disease and 1–3 prior LOT, including with a PI and immunomodulatory drug, with index date of 2016 or later, were identified (Figure 1). Of 82,801 EHR patients with MM, 1597 patients were identified.

Baseline characteristics for patients with lenalidomide‐refractory disease are outlined in Table 1. Median age was 72 years (interquartile range [IQR]: 65–78) for Claims patients and 68 years (IQR: 60–76) for EHR patients. Black patients are represented in the Optum EHR population at 19.2%, consistent with the statistic that 1 in 5 patients diagnosed with MM in the United States is Black [12]. Details of race and ethnicity were not available in the Optum Claims database. In the Optum Claims dataset, 752 (54.4%) patients had received 1 prior LOT, 493 (35.6%) had received 2 prior LOT, and 138 (10.0%) had received 3 prior LOT. In the Optum EHR dataset, 688 (43.1%) patients had received 1 prior LOT, 631 (39.5%) had received 2 prior LOT, and 278 (17.4%) had received 3 prior LOT. Median time since diagnosis was 12 months (IQR: 6–28) for Claims and 15 months (IQR: 7–37) for EHR patients. Time since diagnosis increased with LOT, at 7 months (IQR: 5–16), 19 months (IQR: 10–35), and 27 months (IQR: 15–38) for Claims patients with 1, 2, and 3 prior LOT, respectively. For EHR patients, these values were 7 months (IQR: 5–17), 22 months (IQR: 10–45), and 32 months (IQR: 17–49).

**TABLE 1:** Baseline characteristics for patients with lenalidomide‐refractory disease.

Variables	Optum claims (*N* = 1383)	Optum EHR (*N* = 1597)
Age, median (IQR), y	72 (65–78)	68 (60–76)
Male, *n* (%)	728 (52.6)	857 (53.7)
Race, *n* (%)		
White	N/A	1181 (74.0)
Black	N/A	307 (19.2)
Asian	N/A	24 (1.5)
Other/unknown	N/A	85 (5.3)
Time from diagnosis to T0, median (IQR), months	12.0 (6.0–28.0)	15.0 (7.0–37.0)
Prior LOT, *n* (%)		
1	752 (54.4)	688 (43.1)
2	493 (35.6)	631 (39.5)
3	138 (10.0)	278 (17.4)
Prior stem cell transplant, *n* (%)	172 (12.4)	86 (5.4)
Most common prior treatments, *n* (%)		
Lenalidomide	1383 (100.0)	1597 (100.0)
Bortezomib	1284 (92.8)	1396 (87.4)
Daratumumab	161 (11.6)	208 (13.0)
Carfilzomib	141 (10.2)	248 (15.5)
Ixazomib	105 (7.6)	154 (9.6)
Pomalidomide	59 (4.3)	124 (7.8)
Refractory status, *n* (%)		
Single refractory (IMiD)	458 (33.1)	404 (25.3)
PI + IMiD	835 (60.4)	1064 (66.6)
Anti‐CD38 antibody + IMiD	8 (0.6)	9 (0.6)
Triple refractory^a^	82 (5.9)	118 (7.4)
CCI score, mean (SD)	4.0 (3.3)	3.1 (3.0)
Elixhauser comorbidities at T0 in ≥ 25% of patients, *n* (%)		
Hypertension	1091 (78.9)	1027 (64.3)
Fluid and electrolyte disorders	771 (55.7)	790 (49.5)
Renal failure	633 (45.8)	535 (33.5)
Cardiac arrhythmia	556 (40.2)	572 (35.8)
Coagulopathy	513 (37.1)	487 (30.5)
Deficiency anemia	502 (36.3)	288 (18.0)
Diabetes	485 (35.1)	438 (27.4)
Valvular disease	438 (31.7)	369 (23.1)
Metastatic cancer	413 (29.9)	356 (22.3)
Depression	362 (26.2)	410 (25.7)
Chronic pulmonary disease	361 (26.1)	373 (23.4)
Peripheral vascular disorders	360 (26.0)	233 (14.6)

Abbreviations: CCI, Charlson Comorbidity Index; EHR, electronic health record; IMiD, immunomodulatory drug; IQR, interquartile range; LOT, lines of therapy; N/A, not applicable; PI, proteasome inhibitor; SD, standard deviation; T0, time zero (index date).

^a^ ≥ 1 PI + ≥ 1 IMiD + ≥ 1 anti‐CD38 antibody.

Patients had significant comorbidities, with mean Charlson Comorbidity Index scores [13] of 4.0 (Claims) and 3.1 (EHR). The most prominent Elixhauser comorbidities were hypertension (Claims: 78.9%; EHR: 64.3%), fluid and electrolyte disorders (55.7%; 49.5%), renal failure (45.8%; 33.5%), cardiac arrhythmia (40.2%; 35.8%), and coagulopathy (37.1%; 30.5%).

Therapies for MM used before the index date included lenalidomide (100%), bortezomib (Claims: 92.8%; EHR: 87.4%), daratumumab (11.6%; 13.0%), and carfilzomib (10.2%; 15.5%), among others. Autologous stem cell transplant (ASCT) was used in 12.4% of Claims patients and 5.4% of EHR patients pre‐index. Most patients receiving pre‐index ASCT were younger than 65 years (68 out of 172 [39.5%] with transplant in the Claims cohort and 40 out of 86 [46.5%] in the EHR cohort).

### 3.2 Treatment Patterns for Patients With Lenalidomide‐Refractory Disease

During index LOT, 20% of Claims patients and 16% of EHR patients received ASCT. Approximately half of the patients received doublet or triplet regimens as index treatment (Table 2). Doublet therapy was received by 29.5% of Claims patients and 22.0% of EHR patients; triplet therapy was received by 28.6% and 21.0%, respectively. A large proportion of patients (Claims: 37.5%; EHR: 53.5%) received regimens not classified as doublet, triplet, or quadruplet. The most common treatment regimens given in the index LOT were daratumumab–bortezomib–dexamethasone (DVd) (6.6%; 3.5%), daratumumab–pomalidomide–dexamethasone (DPd) (5.9%; 4.2%), and pomalidomide–dexamethasone (Pd) (5.1%; 3.6%). Daratumumab–carfilzomib–dexamethasone (DKd) was used in 2.4% (Claims) and 0.6% of patients (EHR). Use of National Comprehensive Cancer Network (NCCN)‐recommended treatment regimens (including those that are “preferred” for patients with lenalidomide‐refractory RRMM and other recommended regimens for MM, as per NCCN Guidelines, Multiple Myeloma, 2024) was low and declined further as patients progressed through LOT. For example, in patients with 1 prior LOT in the Claims database, use of these regimens went from 36.5% in LOT 2 (index LOT) to 30.0%, 9.2%, and 3.5% in LOT 3, 4, and 5, respectively. Use of NCCN‐recommended regimens was lower overall in the EHR database, with 19.4%, 25.9%, 5.7%, and 1.7% in LOT 2–5.

**TABLE 2:** Most common index treatment regimens for patients with lenalidomide‐refractory disease.

Treatment regimens, *n* (%)	Optum claims (*N* = 1383)	Optum EHR (*N* = 1597)
Doublet^a^	408 (29.5)	351 (22.0)
Triplet^a^	396 (28.6)	335 (21.0)
Quadruplet^a^	61 (4.4)	57 (3.6)
Other	518 (37.5)	854 (53.5)
Treatment regimens received by ≥ 2% of patients^b^		
Daratumumab–bortezomib–dexamethasone^c^	91 (6.6)	56 (3.5)
Daratumumab–pomalidomide–dexamethasone^c^	81 (5.9)	67 (4.2)
Pomalidomide–dexamethasone	70 (5.1)	58 (3.6)
Daratumumab monotherapy	64 (4.6)	50 (3.1)
Cyclophosphamide–bortezomib–dexamethasone	61 (4.4)	48 (3.0)
Pomalidomide–bortezomib–dexamethasone^c^	43 (3.1)	32 (2.0)
Carfilzomib–pomalidomide–dexamethasone	38 (2.7)	32 (2.0)
Carfilzomib–dexamethasone	33 (2.4)	16 (1.0)
Daratumumab–carfilzomib–dexamethasone	33 (2.4)	9 (0.6)
Bortezomib–melphalan	32 (2.3)	24 (1.5)
Ixazomib–dexamethasone	29 (2.1)	17 (1.1)
Bortezomib–dexamethasone	28 (2.0)	27 (1.7)
Daratumumab–bortezomib–pomalidomide	28 (2.0)	19 (1.2)

Abbreviations: EHR, electronic health record; IMiD, immunomodulatory drug; MM, multiple myeloma; NCCN, National Comprehensive Cancer Network; PI, proteasome inhibitor.

^a^ Doublet regimen defined as any of one PI, IMiD, anti‐CD38, selinexor, belantamab mafodotin, or elotuzumab, with or without steroid; triplet regimen defined as any two of the above drugs, with or without steroid; quadruplet regimen defined as any three of the above drugs, with or without steroid. Steroid use was not always captured in the database as a component of combination regimens; its use was assumed if not reported with other regimen components.

^b^ Dexamethasone (18.3% for Claims and 32.2% for EHR), melphalan (11.3% and 14.3%), and prednisolone (4.8% and 3.4%) were also reported but are not considered monotherapy treatments for MM and were likely captured in the databases as one part of a combination and/or transplant regimen.

^c^ NCCN‐preferred regimen for lenalidomide‐refractory MM.

**FIGURE 2:**
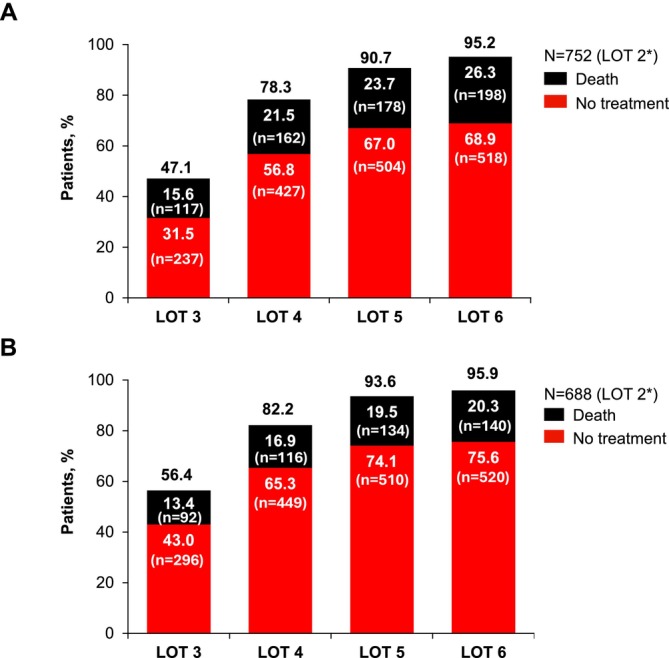
Cumulative attritions in successive LOT after the index LOT (LOT 2) in patients with lenalidomide‐refractory RRMM and 1 prior LOT from the Claims (A) and EHR (B) databases. *Eligible patients who had 1 prior LOT were included in this cumulative attrition analysis. EHR, electronic health records; LOT, lines of therapy; RRMM, relapsed/refractory multiple myeloma.

In the Claims database, the treatment attrition rate between LOT 2 and LOT 3 was 47.1%, including 31.5% of patients who did not have any treatment recorded after LOT 2% and 15.6% of patients who died before LOT 3 (Figure 2). The attrition rate was similar in the EHR database, at 56.4% after LOT 2.

### 3.3 HCRU in Patients With Lenalidomide‐Refractory Disease in the Claims Database

From the start of one LOT until 1 day before the start of the next LOT, the frequency of outpatient visits did not appreciably change with successive LOT (mean number of visits per month ranged from 3.9 to 4.8 across LOT 1 to LOT 4; Table 3). Hospitalizations (including overnight stays) were reported in 35.7%–46.4% of patients across LOT, and among those with hospitalizations, the median aggregate length of stay ranged from 0.42 to 1.04 days per month. The proportions of patients with emergency visits were also similar across LOT (42.3%–46.5%), and the mean number of emergency visits per patient per month ranged from 0.13 to 0.22. The mean number of laboratory visits per patient per month ranged from 1.7 to 2.3 across LOT. Overall, a trend for increased HCRU was not observed as patients went through successive LOT.

**TABLE 3:** HCRU by LOT during the on‐ and off‐treatment periods (Claims database).^a^

	LOT 1	LOT 2	LOT 3	LOT 4
Claims database, *N*	1383	697	300	144
Patients with ≥ 1 outpatient visit, *n* (%)	1282 (92.7)	663 (95.1)	285 (95.0)	133 (92.4)
Outpatient visits PPPM, mean (SD)	4.8 (3.3)	3.9 (2.8)	4.5 (3.0)	4.8 (2.6)
Patients with ≥ 1 inpatient stay, *n* (%)	642 (46.4)	259 (37.2)	107 (35.7)	66 (45.8)
Inpatient stays PPPM, mean (SD)	0.15 (0.47)	0.09 (0.46)	0.14 (0.60)	0.24 (1.07)
Aggregate length of stay PPPM,^b^ median (IQR), days	0.98 (0.35–2.61)	0.42 (0.13–1.45)	0.97 (0.45–2.56)	1.04 (0.40–2.34)
Patients with ≥ 1 ED visit, *n* (%)	610 (44.1)	310 (44.5)	127 (42.3)	67 (46.5)
ED visits PPPM, mean (SD)	0.18 (0.36)	0.13 (0.31)	0.18 (0.36)	0.22 (0.33)
Patients with ≥ 1 laboratory visit, *n* (%)	1251 (90.5)	641 (92.0)	276 (92.0)	127 (88.2)
Laboratory visits PPPM, mean (SD)	2.1 (1.6)	1.7 (1.3)	2.1 (1.4)	2.3 (1.4)

Abbreviations: ED, emergency department; HCRU, health care resource utilization; IQR, interquartile range; LOT, lines of therapy; PPPM, per patient per month; SD, standard deviation.

^a^ From start of LOT until 1 day before start of next LOT.

^b^ Among patients with ≥ 1 hospitalization.

### 3.4 Outcomes in Patients With Lenalidomide‐Refractory Disease

**FIGURE 3:**
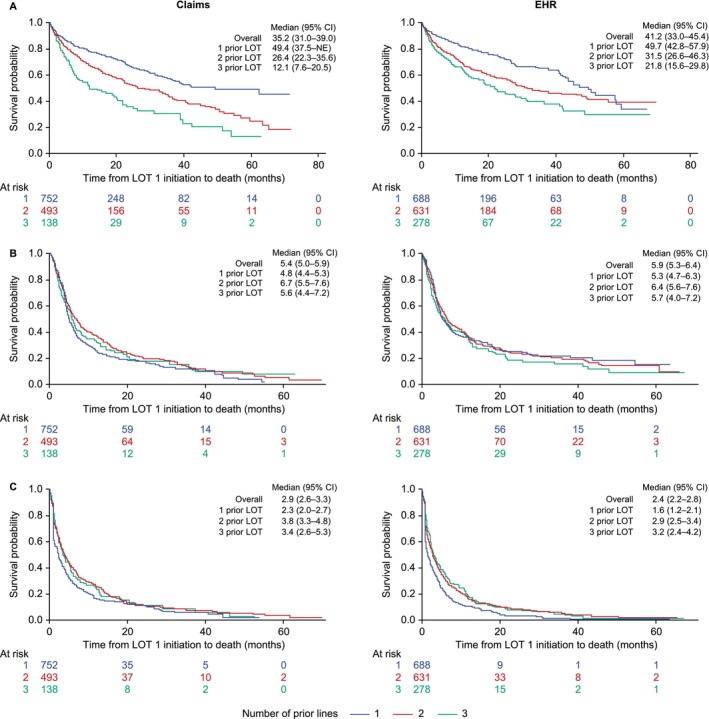
Overall survival (A), time to next treatment or death (B), and time to treatment discontinuation (C) by number of prior LOT for patients with lenalidomide‐refractory RRMM. CI, confidence interval; EHR, electronic health records; LOT, lines of therapy; NE, not estimable; RRMM, relapsed/refractory multiple myeloma.

OS outcomes in all cohorts were poor and tended to decrease with each successive LOT (Figure 3). Median OS was 35.2 months (95% CI, 31.0–39.0) for the Claims cohort and 41.2 months (95% CI, 33.0–45.4) for the EHR cohort. Median OS for 1 prior LOT was 49.4 (Claims: 95% CI, 37.5–not estimable) and 49.7 months (EHR: 95% CI, 42.8–57.9); for 2 prior LOT was 26.4 months (Claims: 95% CI, 22.3–35.6) and 31.5 months (EHR: 95% CI, 26.6–46.3); and for 3 prior LOT was 12.1 months (Claims: 95% CI, 7.6–20.5) and 21.8 months (EHR: 95% CI, 15.6–29.8).

TTNT in the setting of lenalidomide‐refractory disease was short, with a median of 5.4 months (95% CI, 5.0–5.9) for the Claims cohort and 5.9 months (95% CI, 5.3–6.4) for the EHR cohort. Median TTNT was relatively consistent, regardless of the number of prior LOT. Median TTNT for patients with 1, 2, and 3 prior LOT was 4.8 (95% CI, 4.4–5.3), 6.7 (95% CI, 5.5–7.6), and 5.6 months (95% CI, 4.4–7.2), respectively, for Claims patients, and 5.3 (95% CI, 4.7–6.3), 6.4 (95% CI, 5.6–7.6), and 5.7 months (95% CI, 4.0–7.2), respectively, for EHR patients.

Median times to treatment discontinuation were 2.9 months (Claims: 95% CI, 2.6–3.3) and 2.4 months (EHR: 95% CI, 2.2–2.8). TTTD for 1 prior LOT was 2.3 months (Claims: 95% CI, 2.0–2.7) and 1.6 months (EHR: 95% CI, 1.2–2.1); for 2 prior LOT was 3.8 months (Claims: 95% CI, 3.3–4.8) and 2.9 months (EHR: 95% CI, 2.5–3.4); and for 3 prior LOT was 3.4 months (Claims: 95% CI, 2.6–5.3) and 3.2 months (EHR: 95% CI, 2.4–4.2).

### 3.5 Outcomes in Patients With Lenalidomide‐Exposed‐Nonrefractory Disease

Outcomes were also explored for patients who were exposed to lenalidomide but whose disease was lenalidomide nonrefractory for additional context. For this analysis, 679 patients were identified from the Claims database and 960 from the EHR (Table S1). Median OS was 46.1 months (95% CI, 32.6–58.3) for the Claims cohort and 48.4 months (95% CI, 41.1–55.0) for the EHR cohort (Figure S1). Median TTNT was 19.8 months (95% CI, 17.2–24.3) for the Claims cohort and 14.7 months (95% CI, 12.8–17.6) for the EHR cohort. Median TTTD was 10.3 months (95% CI, 7.5–12.6) for the Claims cohort and 6.9 months (95% CI, 6.2–8.4) for the EHR cohort.

## 4 Discussion

The widespread and often chronic use of lenalidomide in earlier settings for treatment of MM has led to an increasing population of patients with lenalidomide‐refractory disease, for whom there is no uniform treatment approach [1, 2, 3, 14]. Our analysis of patients with PI‐exposed, lenalidomide‐refractory disease who have received 1–3 prior LOT provides real‐world evidence around treatment patterns, HCRU, and outcomes for these patients in the time period following US Food and Drug Administration approval of daratumumab in 2015. These data are lacking in the literature for this patient population.

Consensus guidelines recommend a range of treatment options for patients with disease refractory to lenalidomide, allowing for selection based on patient factors, including previous regimens and disease status [1, 2]. Our results reflect this variety and likely reflect ongoing shifts in treatment, with the emerging evidence base supporting triplet combinations. Triplet therapies currently recommended by the NCCN for lenalidomide‐refractory disease (DKd, DVd, DPd, isatuximab–carfilzomib–dexamethasone [IsaKd], isatuximab–pomalidomide–dexamethasone [IsaPd], pomalidomide–bortezomib–dexamethasone [PVd], carfilzomib–pomalidomide–dexamethasone, ixazomib (Ixa)–pomalidomide–dexamethasone, and elotuzumab–pomalidomide–dexamethasone [EloPd]) [1]; venetoclax–bortezomib–dexamethasone and selinexor–bortezomib–dexamethasone are additionally recommended by the European Society for Medical Oncology [2]. Triplet therapy is associated with improved efficacy compared with doublet therapy. Pomalidomide combined with bortezomib–dexamethasone (Vd) significantly increased PFS compared with Vd in a population with many (70%) patients with lenalidomide‐refractory disease [6]. Furthermore, the addition of daratumumab to Vd, carfilzomib–dexamethasone (Kd), or Pd significantly increased PFS, as did the addition of isatuximab to Pd and Kd [7, 15, 16, 17, 18].

Although outcomes are improving in clinical trials with newer regimens, our real‐world data in a more comorbid patient population show they remain suboptimal in real‐world practice across the body of treatment options. TTNT is used as a proxy for PFS; however, TTNT can be longer than PFS due to the time needed to revise the treatment plan after progression has occurred. It is also possible for TTNT to be shorter than PFS if a patient changes treatment due to toxicity before progression occurs. In this analysis, the observed median TTNT (5.4–5.9 months) is shorter than the PFS reported in many clinical studies; for example, subgroup analyses of patients with lenalidomide‐refractory disease and 1–3 prior LOT from randomized, controlled trials have reported median PFS for DVd, PVd, DPd, IsaPd, and DKd of 7.8 months, 9.5 months, 9.9 months, 11.4 months, and 28.1 months, respectively [5, 6, 7, 18, 19]. Real‐world outcomes complement clinical trial data but are generally worse than those seen in controlled clinical trials, as real‐world datasets tend to capture greater clinical diversity within a patient population, including patients with more comorbidities who are typically underrepresented in clinical trials. Indeed, our data showed high rates of hypertension, renal failure, and coagulopathy, consistent with reports of elevated incidences of these comorbidities in real‐world populations with MM [20, 21, 22]. Additionally, although current guideline‐recommended options were well represented in our study, many less effective treatment options were also captured in our data, which would negatively impact TTNT. Use of chemotherapies, steroids, and doublet combinations was prevalent, indicating that real‐world treatment patterns deviate substantially from treatment guidelines. This observation has been made in other analyses of claims data [23, 24, 25]. Approximately a quarter of patients were treated with triplet therapy, and the carfilzomib‐based triplets DKd and IsaKd, which have shown the longest PFS data for triplets in clinical trials [18, 26], were given to just < 3% (DKd) and 0.1% (IsaKd) of patients. The belantamab–bortezomib–dexamethasone combination, recently reported to achieve a 36.6‐month median PFS, was not used in any patients in this dataset [27]. The relatively low use of triplet combinations likely reflects the emergence of triplet therapy during this study period (e.g., combinations were approved for RRMM in the United States in 2015 [KRd, IxaRd], 2016 [DRd, DVd], 2017 [DPd], 2018 [EloPd], 2020 [DKd, IsaPd], and 2021 [IsaKd]), slow uptake of newer therapies in real‐world practice, recent increasing use of triplets in earlier LOT, and the reality that not all patients can tolerate triplet therapy. The time frame of data capture for this analysis predated the approval of cilta‐cel CAR‐T therapy for patients with lenalidomide‐refractory disease and ≥ 1 prior LOT, and the bispecific antibodies teclistamab, elranatamab, and talquetamab are not approved for use in this population at this time. Additionally, the data collected here were until March 2022, and treatment guidelines were updated since then. However, these treatment patterns and outcomes data demonstrate that there is room for improvement in clinical practice. The observation that a majority of patients are not receiving guideline‐recommended combinations indicates a need for additional clinician education or awareness.

Real‐world data present some limitations for analysis. Due to a lack of clinical details, our definition of refractoriness was strictly based on treatment pattern, and it is possible that some patients with lenalidomide‐refractory disease were misclassified as nonrefractory disease. This would have an effect of enriching the lenalidomide‐refractory group with high‐risk patients. Additionally, using treatment change as an indicator of disease progression in real‐world datasets does not account for alternative reasons for treatment change (e.g., suboptimal response or inability to tolerate therapy).

Our analyses demonstrated that TTNT and TTTD are short for patients with lenalidomide‐refractory disease, suggesting patients moved quickly to their next treatment or may have died before receiving subsequent treatment. Attrition rates were high, with approximately 50% of patients who received LOT 2 lost before receiving LOT 3. This is consistent with attrition rates reported from analyses of other claims‐based databases, which range from 31% to 46% between LOT 2 and LOT 3 [23, 28]. Our rates are higher than those reported by the Canadian Myeloma Research Group [29], possibly due to the limitation of claims data not capturing remission off treatment. However, our data also show that OS was short, at 35.2–41.2 months overall. Together, these results support those from recent reports of treatment outcomes for patients with lenalidomide‐refractory disease from other real‐world data sources, where patients had poor PFS and moved quickly through available therapies [23, 24, 25].

The unmet need in patients with lenalidomide‐refractory MM is highlighted by the comparatively better outcomes seen in patients who were exposed to lenalidomide but whose disease was nonrefractory. In this group, the median TTNT of 19.8 and 14.7 months for the Claims and EHR cohorts, respectively, was numerically longer than the medians of 5.4 months and 5.9 months observed for lenalidomide‐refractory disease. This underscores the importance of evaluating new therapies in the population with lenalidomide‐refractory disease.

The use of TTNT data as a proxy for PFS makes it difficult to directly compare with PFS reported in clinical trials and represents a limitation of this study. The landscape of regulatory approvals for various treatment combinations evolved over the study period, potentially resulting in underestimation of current use practices. Also, the use of steroids may not be fully captured in the databases as a component of combination therapies. Because most agents (e.g., PIs, immunomodulatory drugs) are rarely used in isolation without adding a steroid, the use of steroids in combination therapies has been assumed, unless a treatment was specified as monotherapy (i.e., daratumumab monotherapy). Real‐world data may also be influenced by several clinical, economic, equity, and social factors, such as travel burden, caregiver burden, access, and others [30, 31, 32]. In addition, the EHR database reflects in‐network utilization only; care sought out of network is not captured. This analysis included patients with commercial and Medicare insurance coverage, and the results may not be generalizable to the overall population of US patients with MM, including those on Medicaid. Nevertheless, this study comprehensively evaluated patient outcomes in a representative, real‐world population. Finally, there is some overlap of patients in the two databases and patients were not double counted based on some of their baseline and demographic characteristics; however, not all double‐counted patients could be identified, and therefore the analyses of each database were kept separate.

HCRU data on US patients with lenalidomide‐refractory disease after 1–3 LOT have not been previously reported. Studies in patients with more advanced disease, including patients who are triple‐class exposed and those with ≥ 4 LOT, show similar levels of utilization as the present population [33, 34, 35, 36, 37].

In conclusion, this real‐world analysis of patients with lenalidomide‐refractory MM in the time period since the introduction of daratumumab adds to the limited data that are currently available on this population and provides real‐world benchmarks for comparison with novel therapies in similar populations. Our results demonstrate that outcomes for patients with lenalidomide‐refractory disease are poor, even early in their treatment journey. Their rapid progression through therapies highlights the need for improved patient care with currently available therapies as well as new therapeutic options to improve outcomes in this difficult‐to‐treat patient population.

## Author Contributions

**Binod Dhakal:** conceptualization (supporting), formal analysis (supporting), writing – original draft (equal), writing – review and editing (equal). **Hermann Einsele:** conceptualization (supporting), formal analysis (supporting), writing – original draft (equal), writing – review and editing (equal). **Jordan M. Schecter:** conceptualization (lead), formal analysis (supporting), writing – original draft (equal), writing – review and editing (equal). **William Deraedt:** conceptualization (lead), formal analysis (supporting), writing – original draft (equal), writing – review and editing (equal). **Nikoletta Lendvai:** conceptualization (lead), formal analysis (supporting), writing – original draft (equal), writing – review and editing (equal). **Ana Slaughter:** conceptualization (lead), formal analysis (supporting), writing – original draft (equal), writing – review and editing (equal). **Carolina Lonardi:** conceptualization (lead), formal analysis (supporting), writing – original draft (equal), writing – review and editing (equal). **Sandhya Nair:** conceptualization (lead), data curation (equal), formal analysis (lead), methodology (equal), writing – original draft (equal), writing – review and editing (equal). **Nirosha Elsem Varghese:** conceptualization (lead), data curation (equal), formal analysis (lead), methodology (equal), writing – original draft (equal), writing – review and editing (equal). **Jianming He:** conceptualization (lead), data curation (equal), formal analysis (lead), methodology (equal), writing – original draft (equal), writing – review and editing (equal). **Akshay Kharat:** conceptualization (lead), data curation (equal), formal analysis (lead), methodology (equal), writing – original draft (equal), writing – review and editing (equal). **Seina Lee:** conceptualization (lead), data curation (equal), formal analysis (lead), methodology (equal), writing – original draft (equal), writing – review and editing (equal). **Patricia Cost:** conceptualization (lead), data curation (equal), formal analysis (lead), methodology (equal), writing – original draft (equal), writing – review and editing (equal). **Ravi Potluri:** formal analysis (lead), methodology (equal), writing – original draft (equal), writing – review and editing (equal). **Mythili Koneru:** conceptualization (lead), writing – original draft (equal), writing – review and editing (equal). **Nitin Patel:** conceptualization (lead), writing – original draft (equal), writing – review and editing (equal). **Erika Florendo:** conceptualization (lead), writing – original draft (equal), writing – review and editing (equal). **Paula Rodriguez‐Otero:** conceptualization (supporting), writing – original draft (equal), writing – review and editing (equal). **Kwee Yong:** conceptualization (supporting), writing – original draft (equal), writing – review and editing (equal).

## Ethics Statement

The data sources used in this study (i.e., Optum Claims and EHR databases) contain de‐identified patient datasets and are fully compliant with the Health Insurance Portability and Accountability Act (HIPAA).

## Consent

The authors have nothing to report.

## Conflicts of Interest

Binod Dhakal has served in a consulting or advisory role for Amgen, GlaxoSmithKline, Johnson & Johnson, Natera, Sanofi, and Takeda; has received honoraria from Celgene, GlaxoSmithKline, Karyopharm Therapeutics, and Sanofi; and has received research funding from Amgen, GlaxoSmithKline, and Johnson & Johnson. Hermann Einsele has served in a consulting or advisory role for Amgen, Bristol Myers Squibb, Celgene, Johnson & Johnson, Novartis, and Takeda; has received travel funding from Amgen, Bristol Myers Squibb, Celgene, Johnson & Johnson, and Takeda; has received honoraria from Amgen, Bristol Myers Squibb, Celgene, Johnson & Johnson, Novartis, and Takeda; and has received research funding from Amgen, Bristol Myers Squibb, Celgene, and Johnson & Johnson. Jordan M. Schecter, Ana Slaughter, Sandhya Nair, Nikoletta Lendvai, and Patricia Cost are employed by and own stock in Johnson & Johnson. William Deraedt is employed by, owns stock in, and has received patent royalties from Johnson & Johnson. Carolina Lonardi, Nirosha Elsem Varghese, and Seina Lee are employed by Johnson & Johnson. Akshay Kharat owns stock in and was employed by Johnson & Johnson at the time the study was conducted and is currently employed with Boehringer Ingelheim Pharmaceuticals Inc. Jianming He is employed by and owns stock in Johnson & Johnson and has an immediate family member employed by Bristol Myers Squibb. Ravi Potluri is employed by Putnam Associates. Mythili Koneru, Nitin Patel, and Erika Florendo are employed by Legend Biotech USA Inc. Paula Rodriguez‐Otero has served in a consulting or advisory role for BeiGene, GlaxoSmithKline, Sanofi, and Takeda Oncology and has received research funding from AbbVie, Amgen, Ascentage Pharma, AstraZeneca, Bristol Myers Squibb, Cellectar Biosciences, GlaxoSmithKline, Johnson & Johnson, Pharmacyclics, and Xencor. Kwee Yong has served in a consulting or advisory role for Johnson & Johnson; has participated in speakers' bureaus for Amgen, Sanofi, and Takeda; has received honoraria from Amgen, Johnson & Johnson, Sanofi, and Takeda; and has received research funding from Amgen, Autolus, Johnson & Johnson, Sanofi, and Takeda.

## Supporting information


**Table S1:** Baseline characteristics for patients with lenalidomide‐exposed‐not‐refractory disease.
**Figure S1:** Overall survival (A), and time to next treatment or death (B) for patients with lenalidomide‐exposed‐not‐refractory disease. CI, confidence interval; EHR, electronic health records; LOT, lines of therapy.

## Data Availability

The data sharing policy of Janssen Pharmaceutical Companies of Johnson & Johnson is available at https://www.jnj.com/clinical‐trials/transparency. These data were made available by Optum Clinformatics Data Mart and used under license for the current study and are not publicly available. Other researchers should contact https://optum.com.
