# Supplementary material for: Real‐World Treatment Patterns and Outcomes in Patients With Relapsed/Refractory Multiple Myeloma and 1–3 Prior Lines of Therapy: Optum Database
Source: Cancer Med. 2025 Jul 28;14(15):e71093. doi: 10.1002/cam4.71093 (PMC12301936; doi:10.1002/cam4.71093)

# **SUPPLEMENTARY MATERIAL**

# **Real-World Treatment Patterns and Outcomes in Patients With Relapsed/Refractory Multiple Myeloma and 1–3 Prior Lines of Therapy: Optum Database**

Binod Dhakal, MD^1^, Hermann Einsele, MD^2^, Jordan M. Schecter, MD^3^, William Deraedt, MSc^4^, Nikoletta Lendvai, MD, PhD^3^, Ana Slaughter, PhD^5^, Carolina Lonardi, PharmD^6^, Sandhya Nair, PhD^4^, Nirosha Elsem Varghese, PhD^4^, Jianming He, PhD^7^, Akshay Kharat, PhD^8^, Seina Lee, PharmD, MS^7^, Patricia Cost, MS^7^, Ravi Potluri, MBA^9^, Mythili Koneru, MD, PhD^10^, Nitin Patel, BM BCh^10^, Erika Florendo, MSN^10^, Paula Rodriguez-Otero, MD^11^, Kwee Yong, PhD^12^

^1^Medical College of Wisconsin, Milwaukee, Wisconsin, USA. ^2^Universitätsklinikum Würzburg, Medizinische Klinik und Poliklinik II, Würzburg, Germany. ^3^Johnson & Johnson, Raritan, New Jersey, USA. ^4^Johnson & Johnson, Beerse, Belgium. ^5^Cilag GmbH International, Zug, Switzerland. ^6^Johnson & Johnson, Buenos Aires, Argentina. ^7^Johnson & Johnson, Raritan, New Jersey, USA. ^8^Johnson & Johnson, Horsham, Pennsylvania, USA. ^9^Putnam Associates, New York, New York, USA. ^10^Legend Biotech USA Inc., Somerset, New Jersey, USA. ^11^Clínica Universidad de Navarra, CIMA, CIBERONC, IDISNA, Pamplona, Spain. ^12^University College London Cancer Institute, London, UK.

**Corresponding author:** Dr. Binod Dhakal; **Email**: [bdhakal@mcw.edu](mailto:bdhakal@mcw.edu)

## **Supplementary Table 1.** Baseline characteristics for patients with lenalidomide-exposed-not-refractory disease.

| **Variables** | **Optum Claims**  **(*N* = 679)** | **Optum EHR**  **(*N* = 960)** |
| --- | --- | --- |
| Age, years, median (IQR) | 73 (64–79) | 68 (60–76) |
| Male, *n* (%) | 350 (51.5) | 515 (53.6) |
| Months from diagnosis to index date, median (IQR) | 19 (9–38) | 24 (9–49) |
| Number of prior LOT, *n* (%) |  |  |
| 1 | 442 (65.1) | 500 (52.1) |
| 2 | 195 (28.7) | 344 (35.8) |
| 3 | 42 (6.2) | 116 (12.1) |
| Stem cell transplant (before index date), *n* (%) | 158 (23.3) | 65 (6.8) |
| Refractory status, *n* (%) |  |  |
| PI | 197 (29.0) | 344 (35.8) |
| Anti-CD38 antibody | 1 (0.1) | 3 (0.3) |
| CCI score, mean (SD) | 3.9 (3.1) | 2.7 (3.0) |
| Elixhauser comorbidities at index, *n* (%) |  |  |
| Hypertension | 524 (77.2) | 570 (59.4) |
| Fluid and electrolyte disorders | 335 (49.3) | 378 (39.4) |
| Renal failure | 299 (44.0) | 284 (29.6) |
| Cardiac arrhythmia | 274 (40.4) | 316 (32.9) |

Abbreviations: CCI, Charlson Comorbidity Index; EHR, electronic health records; IQR, interquartile range; IMiD, immunomodulatory drug; LOT, lines of therapy; PI, proteasome inhibitor; SD, standard deviation.

## **Supplementary Figure 1** Overall survival (A), and time to next treatment or death (B) for patients with lenalidomide-exposed-not-refractory disease. CI, confidence interval; EHR, electronic health records; LOT, lines of therapy.


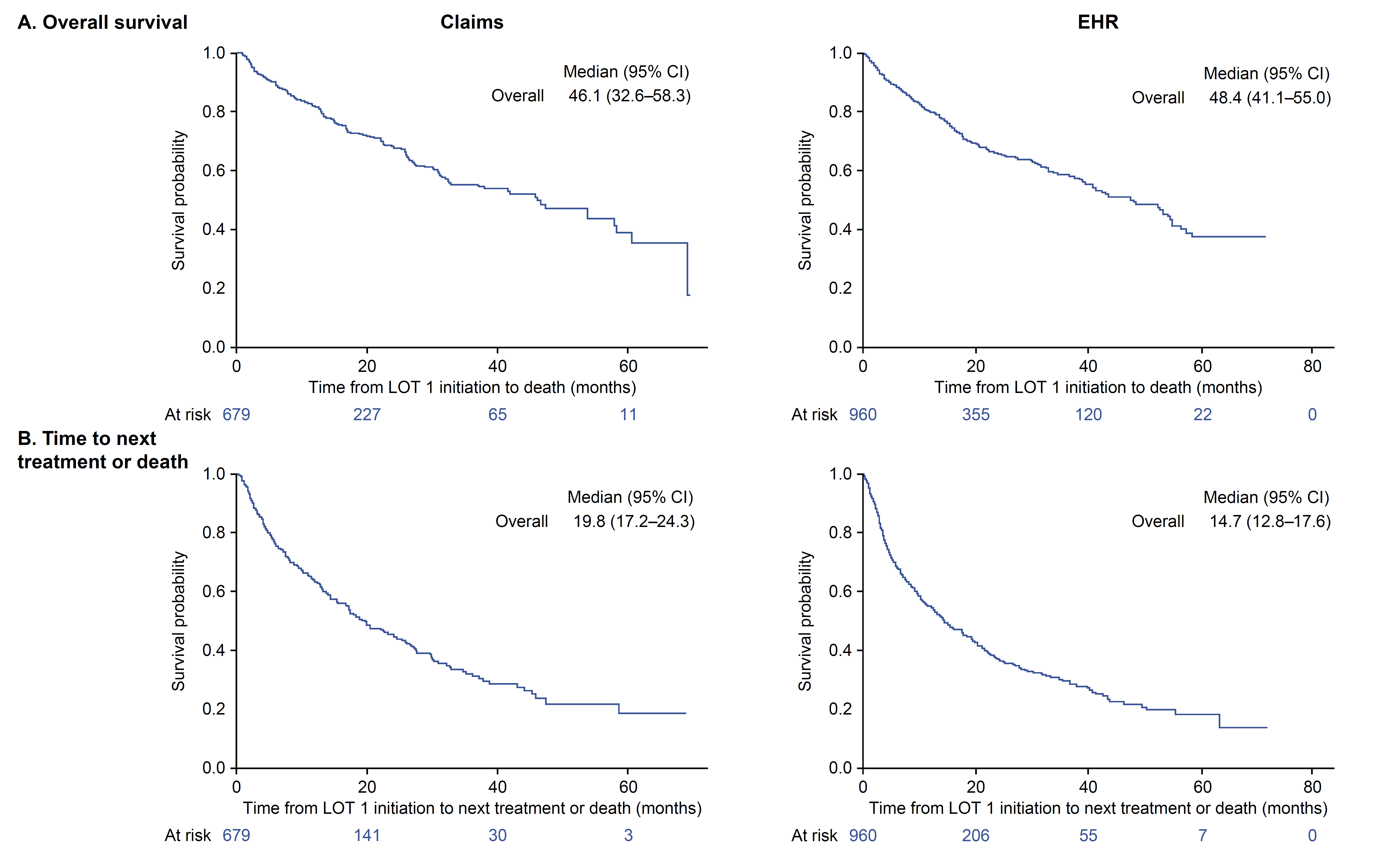

Supplement: Supplementary file 1 — Table S1: Baseline characteristics for patients with lenalidomide‐exposed‐not‐refractory disease. Figure S1: Overall survival (A), and time to next treatment or death (B) for patients with lenalidomide‐exposed‐not‐refractory disease. CI, confidence interval; EHR, electronic health records; LOT, lines of therapy. [file CAM4-14-e71093-s001.docx]
